# 

**Published:** 1916-08-19

**Authors:** 


### LITERARY INTELLIGENCE.

In Mr. E. Lipson's "Europe in the Nineteenth Century," which is about to be published by Messrs. A. and C. Black, is given a concise and connected account—analytical rather than narrative—of the development of the chief European States during the nineteenth century. A chapter on Russia traces the history of the Reform movement down to the present day, and a chapter on "The Concert of Europe" describes the attempts made after the fall of Napoleon to establish a European Confederation. In the last chapter an attempt is made to elucidate the antecedents and underlying motives of the present war.

### Matrons' and Sisters' Appointments.

Beverley Dispensary and Hospital.—Miss Letitia A. Coulson has been appointed matron. She was trained at Manchester Royal Infirmary, and was later night superintendent at the North Staffordshire Infirmary, Stoke-on-Trent.

Ilford Isolation Hospital, Chadwell Heath.—Miss F. Poynton has been appointed assistant matron. She was trained at the Royal Hants County Hospital, Winchester, and has since been sister at Grimsby and Hull Sanatoriums and at the Skipton Isolation Hospital.

EcclesHall Bierlow Infirmary.—Miss Eliza Anne Walker has been appointed home sister. She was trained at Bagthorpe Infirmary, Nottingham, where she has since been ward sister.

Ainsworth Sanatorium, near Bolton.—Miss Clara A. Moseley has been appointed sister. She was trained at Bury Union Hospital, where she has since been staff nurse.

**NOTE.—Particulars of Appointments for insertion in this column will be welcomed.**

### Personalia.

Out of eighty applicants Mr. Charles Keir Alexander, of Aberdeen, has been appointed treasurer of the Montrose Royal Asylum.

Dr. Agnes W. Andrew has been appointed assistant medical officer at the Leeds Public Hospital, Seacroft, at a salary of £250 a year.

Mr. S. L. Buatia, B.A. Cantab., M.R.C.S., L.R.C.P., has been appointed ophthalmic house surgeon at St. Thomas's Hospital.

Captain G. O. Maw, who was house surgeon at the Metropolitan Hospital, London, until he joined the R.A.M.C. early this year, has died from wounds received while attending the wounded in France.

Mr. Ernest Sanger has been appointed a member of the London Mental Hospitals Committee.

### Gifts to Hospitals.

The Victoria Hospital, Bournemouth, receives £1,000 under the will of Mr. Frank Wareham, of Bournemouth, who died on March 18. The same institution receives £100 under the will of Miss E. H. Southby, who died on April 1.

Mr. David Mackenzie Mackay, of Hampstead, who died on June 27, left £500 each to the Ross Memorial Hospital, Dingwall, the Hampstead General Hospital, and the Hospital and Home for Incurable Children, Hampstead.

Towards the cost of the rebuilding of the hospital, the Chelsea Hospital for Women has received donations of £1,000 from two anonymous donors to establish memorial beds in the new hospital, £50 from the Goldsmiths' Company, and £250 from the Rev. Perceval Laurence.

Under the will of the late Mrs. S. L. Geldart, widow of Canon Geldart, of Kirk Deighton, the following allocations have been made under the discretionary powers of the Treasury Solicitor: York County Hospital, £1,000; Leeds General Infirmary, £3,600; Bradford Royal Infirmary, £2,000; and the Chelsea Hospital for Women, £1,600.

Rates of Subscription: Twelve months, 6/6; Six months, 4/-; Three months, 2/-, post free to any address in the United Kingdom. Abroad, Twelve months, 8/8; Six months, 5/-; Three months, 2/6, post free. The Hospital "Index" to Volume LIX., October 1915 to March 1916, is now ready, price 1½d. per copy, post free. Address The Manager, "The Hospital," The Hospital Building, 28/29 Southampton Street, Strand, London, W.C.

**NATIONAL RELIEF FUND.**

**Treasurer—H.R.H. The Prince of Wales.**

**Will every Reader of "THE HOSPITAL" send at least 1s.**

To H.R.H. THE PRINCE OF WALES,
BUCKINGHAM PALACE, LONDON

*I beg to enclose £ s. d. as a donation to the National Relief Fund.*

*Name* ..........................................

*Address* ..........................................

..........................................

The envelope containing this coupon need not be stamped.

